# Erratum for “The relationship between radiographic disc calcification score and FGF4L2 genotype in dachshunds”

**DOI:** 10.1111/jvim.70061

**Published:** 2025-03-13

Stacey Sullivan , David Redden , Froydis Hardeng , Malin Sundqvist , Michelle Kutzler J Vet Intern Med. 2025; 39(1). 10.1111/jvim.17281.PMC1166596039715441

In the article cited above, in the Results section of the Abstract the second sentence erroneously states, “1 or 2 FGF4L2 copies.” This should state “0 or 1 FGF4L2 copies.” This concept is correctly stated in the main body of the article.

